# Ketamine in psychiatry: Ethical imperatives in harnessing a controversial yet promising therapy

**DOI:** 10.1177/00048674251396009

**Published:** 2025-12-11

**Authors:** Adem T Can, Jim Lagopoulos, Paul B Fitzgerald, Neil W Bailey, Megan Dutton

**Affiliations:** 1 Thompson Brain & Mind Healthcare, Sunshine Coast, QLD, Australia; 2 School of Medicine and Psychology, The Australian National University, Canberra, ACT, Australia

**Keywords:** Ketamine, depression, ethics, antidepressants

## Abstract

Ketamine has emerged as a rapid-acting intervention for treatment-resistant psychiatric disorders, generating both enthusiasm and unease. While evidence demonstrates robust antidepressant, anxiolytic and anti-suicidal effects, ketamine also carries risks, including dissociation, dependence and uncertain long-term safety. Its reputation as a recreational drug further complicates clinical adoption, fostering stigma and regulatory caution. In this article, we consider ketamine’s psychiatric use through the lens of medical ethics, structured around the principles of autonomy, beneficence, non-maleficence and justice. We argue that while ketamine should be embraced as a legitimate psychiatric therapy, its application must be grounded in rigorous ethical practice, supported by regulation and research, and shielded from both undue dismissal and premature over-promotion.

## Introduction

For more than six decades, ketamine has been used safely in anaesthesia, analgesia and emergency medicine. In recent years, however, its role has expanded into psychiatry, where sub-anaesthetic doses have been shown to produce rapid improvements in mood, anxiety and suicidality (Walsh et al., 2021). For individuals facing treatment-resistant depression or acute suicidal ideation, ketamine can represent a critical lifeline when conventional treatments have failed (Zolghadriha et al., 2024).

Yet ketamine’s promise is accompanied by challenges. The durability of its effects is limited, long-term safety remains uncertain, and its history as a recreational drug, popularly known as ‘Special K’ contributes to stigma that shapes public perception, clinical decision-making and health policy (Beerten et al., 2023).

The ethical dilemmas surrounding ketamine extend beyond safety. They encompass issues of informed consent, the risk of misuse, inequitable access, and the balance between clinical innovation and patient protection. In this article, we explore these issues through the framework of biomedical ethics, advocating for a balanced, ethically sound approach to ketamine in psychiatry.

## Ketamine’s clinical potential and controversy

Ketamine distinguishes itself from conventional antidepressants in two important respects: speed and efficacy in otherwise refractory cases. In contrast to antidepressants (which take weeks to show effect), randomised controlled as well as open-label trials demonstrate reductions in depressive symptoms within hours of administration (oral, intranasal or intravenous [IV]), with additional benefits for anxiety and suicidal ideation (Can et al., 2021, 2022, 2023; Singh et al., 2017).

However, these benefits are often transient, with relapse sometimes occurring within days or weeks without repeated dosing (Short et al., 2018). Long-term effects remain under investigation but have not been nearly as well demonstrated as the effects demonstrated in short-term trials (Massaneda-Tuneu et al., 2026; Nikolin et al., 2023). There are also concerns about potential cognitive impairment from ketamine, urinary toxicity and the risk of tolerance or dependence associated with longer-term use (Carroll et al., 2024). There is also some uncertainty as to whether ketamine’s benefits may be greater or not when embedded in a psychotherapeutic framework. In this regard, a recent trial (Lii et al., 2023) demonstrated that ketamine’s antidepressant effects may depend on psychotherapeutic context or expectancy-related mechanisms rather than being purely pharmacological. These findings highlight the need for future research to examine integrative approaches that deliberately incorporate the psychological and experiential dimensions of ketamine administration.

Ketamine’s dual identity, as both a therapeutic agent and a substance of misuse, complicates its clinical adoption. In addition to its therapeutic role, it is a controlled substance with a history of recreational misuse. This association contributes to a stigma, which can deter patients from accepting treatment and fuel scepticism among clinicians and policymakers (Zhang et al., 2017). At the same time, ketamine’s reputation as a recreational drug poses a different risk, as some patients may be drawn to treatment primarily for its psychoactive effects rather than its therapeutic potential, raising concerns about suitability and the possibility of inappropriate use. Thus, clinical application must navigate both biological risk and sociocultural perception. These clinical complexities underscore the need for an ethically grounded framework to guide ketamine’s use in psychiatry.

## Ethical principles applied to ketamine

### Autonomy

Respect for autonomy requires that patients make informed, voluntary choices about their care. In ketamine treatment, this entails clear disclosure of:

- The rapid but short-lived nature of benefits.
- Known risks, including dissociation, blood pressure elevation, nausea and transient perceptual changes.
- Uncertainty regarding long-term safety and efficacy.
- The experimental status of some off-label psychiatric uses.

Patients should receive information in accessible language, with sensitivity to cultural and educational differences (Sathappan and Yudkoff, 2024). However, as Mathai et al. (2022) observed in their review of ketamine treatment information sheets, the quality and breadth of patient-facing materials are often suboptimal, suggesting that many patients may not be receiving sufficiently comprehensive or balanced information. Where decision-making capacity is impaired by severe depression or suicidality, clinicians should involve proxies or guardians. Autonomy also requires that patients may refuse or discontinue ketamine without coercion, even when clinicians believe it may help.

An additional challenge arises during acute administration. Once ketamine is given, its psychoactive effects cannot be discontinued until they naturally subside, usually over 30–90 minutes. During this period, some patients may experience distressing dissociation, perceptual changes or anxiety and may wish to stop, but immediate cessation is not possible. This limitation underscores the importance of thorough pre-treatment consent, careful dose titration and continuous clinical monitoring. Current guidelines recommend subcutaneous or intravenous administration only in supervised healthcare settings with trained staff present to manage potential adverse events (Australian and New Zealand College of Anaesthetists Faculty of Pain Medicine, 2017; Hussain et al., 2025; WA Country Health Service, 2020).

### Beneficence

Beneficence obliges clinicians to act in the best interest of patients. In psychiatry, ketamine exemplifies beneficence by offering relief where other interventions have failed. It can rapidly alleviate otherwise intractable suffering, restore functioning and in cases of suicidality, potentially save lives (Kew et al., 2023).

Application of beneficence also involves recognising ketamine’s potential to catalyse engagement with broader therapeutic modalities. Temporary relief from severe depression or suicidal ideation can create a ‘therapeutic window’ in which patients are more able to participate in psychotherapy, safety planning or functional recovery (Wilkinson et al., 2018). Trials in post-traumatic stress disorder (PTSD) and substance use similarly suggest ketamine may enhance responsiveness to trauma-focused or behavioural interventions (Dakwar et al., 2019; Feder et al., 2014). Real-world studies of ketamine-assisted psychotherapy further support its role as a facilitator of durable change when integrated with psychological treatment (Dore et al., 2019).

Careful patient selection is essential, with dosing tailored to individual needs and ketamine embedded within a broader therapeutic plan. Pharmacological treatment should be accompanied by psychotherapy, lifestyle interventions and close monitoring, with regular evaluation to confirm ongoing benefit (Singh et al., 2017). Patients too impaired to engage in therapy may gain from ketamine’s rapid but temporary relief, creating a window for participation. In contrast, those with substance misuse histories or seeking its psychoactive effects may be poor candidates. Screening for comorbidities, motivation and capacity for follow-up helps identify individuals most likely to translate short-term relief into sustained engagement in therapy and daily functioning.

Beneficence also entails avoiding therapeutic nihilism. To withhold ketamine solely due to stigma or regulatory hesitancy may deny patients access to potentially transformative care. Where conventional treatments have failed, even temporary relief can restore hope and function, creating opportunities for further recovery. Ethically, clinicians have a duty to balance caution with innovation, ensuring that promising treatments are neither prematurely dismissed nor offered without appropriate safeguards.

### Non-maleficence

The principle of non-maleficence, ‘first, do no harm’, is critical in guiding ketamine use. Potential harms include acute adverse effects (dissociation, dizziness, cardiovascular instability), long-term risks (cognitive impairment, urinary toxicity) and the possibility of dependence (Short et al., 2018).

Ethically sound practice requires:

- Administration only in controlled clinical settings.
- Comprehensive pre-treatment screening, including for substance use and cardiovascular disease.
- Limiting treatment to the lowest effective dose and appropriate frequency.
- Transparent discussion of the unknowns surrounding long-term use.

Equally, non-maleficence cautions against over-promising. Framing ketamine as a panacea risks undermining patient trust when its effects prove transient. Ethical communication must balance hope with realism.

Past experience in Australia underscores these concerns. In the mid-2010s, private ketamine services emerged that offered off-label treatment for psychiatric conditions but were subsequently closed following regulatory scrutiny. Reports highlighted inadequate clinical oversight, provision of take-home ketamine and marketing practices that outpaced the available evidence (Nulley, 2015; Worthington, 2015). Such episodes illustrate how insufficient safeguards can expose patients to harm and erode public trust. They reinforce the ethical imperative that ketamine be administered only within rigorously regulated and evidence-based frameworks (Short et al., 2018).

### Justice

Justice demands fairness in access and distribution of healthcare resources. At present, ketamine treatment is often limited to private clinics in urban centres, with costs borne out-of-pocket by patients. However, there are some public services in Australia (e.g. Royal Prince Alfred Hospital, Gold Coast University Hospital) who offer ketamine publicly with the treatment subsidised by the public mental health system (Thornton et al., 2024). This creates inequities, as lower-income individuals and those in rural regions are excluded (Peskin et al., 2023). While ketamine itself is listed on the Pharmaceutical Benefits Scheme for certain indications, there is no funding provision for the essential supervision, monitoring and clinical support required during administration for psychiatric use. This gap means that even if the drug cost were subsidised, the substantial service delivery costs remain out-of-pocket, perpetuating inequity. Policymakers should consider subsidising ketamine treatment within public mental health frameworks to reduce socioeconomic disparities.

Transparent eligibility criteria are essential to guide patient selection, ensuring that those most likely to benefit from ketamine treatment are prioritised. Rather than blanket exclusions, nuanced assessments should determine suitability, particularly for individuals with a history of substance misuse. Anti-stigma initiatives and targeted clinician training can help prevent unjustified exclusion of such patients while also recognising that past misuse may introduce unique vulnerabilities, including heightened risk of dependence or inappropriate use. Structured screening protocols, harm-reduction strategies and integration of psychosocial supports can mitigate these risks. This balanced approach promotes equity of access while safeguarding patient safety and upholding ethical standards in clinical practice.

Justice also requires careful allocation of healthcare resources. While ketamine shows promise, it must not eclipse investment in other evidence-based therapies. Cost-effectiveness analysis and balanced integration into psychiatric care pathways are essential (Price et al., 2022).

## Regulatory and research imperatives

The absence of standardised global guidelines undermines the ethical delivery of ketamine treatment. In Australia, emerging national guidelines, such as those developed by the Royal Australian and New Zealand College of Psychiatrists (RANZCP; Hussain et al., 2025), provide a preliminary framework for safe and responsible clinical use of ketamine, but their adoption remains variable across services. Current practice varies not only in dose and frequency but also in mode of administration, with intravenous, intramuscular and intranasal routes all employed across different clinics. Further inconsistency arises between the use of racemic ketamine (currently offered off-label in Australia) and Esketamine, each with differing evidence bases, regulatory approvals and cost implications. Notably, the TREK Study which is a multi-centre Australian randomised, prospective, rater-blinded trial is currently comparing the effectiveness, safety, acceptability and cost-effectiveness of intranasal esketamine vs intravenous racemic ketamine in adults with treatment-resistant depression (ClinicalTrials.gov Identifier: NCT06278779). Findings from this trial are expected to help inform future clinical standards and improve treatment consistency. However, hitherto such variability contributes to unpredictable outcomes and complicates comparisons across studies and services. The development of comprehensive, evidence-based protocols that address these modalities is urgently needed to ensure safe, equitable and consistent care.

Equally pressing is the need for long-term research. Most studies focus on short-term outcomes; few address durability, safety or effects of chronic use. Large-scale, longitudinal trials are essential to inform best practice. Research should include diverse populations to ensure findings are generalisable across cultures and socioeconomic groups.

Without regulatory oversight and scientific clarity, ketamine risks either over-commercialisation or unwarranted restriction, both ethically problematic extremes.

## Reconciling stigma and science

Stigma remains a profound barrier. Ketamine’s history as a recreational drug continues to shape perceptions, leading to scepticism from clinicians, hesitancy from patients and reluctance from policymakers (Walsh et al., 2022). This stigma undermines autonomy, beneficence and justice by discouraging informed choice, limiting therapeutic opportunities and entrenching inequities.

Addressing stigma requires proactive communication. Clinicians must educate patients and the public about ketamine’s legitimate medical use, its evidence base and its risks. Media framing should be monitored to avoid sensationalism that conflates clinical practice with illicit misuse (Zhang et al., 2017). Over time, normalising ketamine within psychiatry may mirror the trajectory of other once-stigmatised therapies, such as ECT, which regained legitimacy through rigorous evidence and ethical application. Like ECT which overcame early stigma through rigorous evidence and ethical reappraisal, ketamine’s legitimacy depends on sustained scientific and ethical engagement. It should be noted, however, that this perspective on ECT is not universally supported.

## Discussion

Ketamine epitomises the ethical challenges of modern psychiatry, navigating between hope and hype, potential and peril. Its therapeutic benefits for treatment-resistant depression are substantial, yet its risks and stigma remain real.

The ethical framework of autonomy, beneficence, non-maleficence and justice provides a roadmap for responsible adoption. Patients must be empowered through informed consent, clinicians must act in patients’ best interests while avoiding harm, and health systems must ensure equitable access.

The greatest danger lies in the extreme positions, which either dismiss ketamine due to its illicit past or embrace it uncritically as a panacea. Both approaches neglect the nuanced, ethically grounded practice required to safeguard patients and advance psychiatry responsibly. Ongoing ethical discourse is essential to navigate emerging challenges as ketamine’s role in psychiatry evolves.

## Conclusion

Ketamine treatment in psychiatry is neither a miracle cure nor a dangerous indulgence. It is a powerful psychiatric intervention that demands careful, ethical integration into practice. To ignore ketamine’s potential because of its history is to abandon patients in need, while to adopt it without safeguards is to risk exploitation and harm.

The ethical imperative is clear: psychiatry must embrace ketamine with humility, caution and commitment to patient welfare. By embedding ketamine’s use within a framework of ethical principles, supported by research and regulation, psychiatry can transform a drug of controversy into a therapy of compassion and justice.
